# Examining the effect of logistics service quality on customer satisfaction and re-use intention

**DOI:** 10.1371/journal.pone.0286382

**Published:** 2023-05-31

**Authors:** Xiaofang Lin, Abdullah Al Mamun, Qing Yang, Mohammad Masukujjaman

**Affiliations:** 1 School of Business, Nantong Institute of Technology, Nantong City, Jiangsu Province, China; 2 UKM—Graduate School of Business, Universiti Kebangsaan Malaysia, UKM Bangi, Selangor Darul Ehsan, Malaysia; Universidad Central de Chile, CHILE

## Abstract

For logistics service providers (LSPs), improving customer satisfaction and obtaining customer re-use intention are key to gaining sustainable competitive advantages and success. Logistics service quality (LSQ) is a concern for logistics service providers, retailers, and customers. The proposed model, which is based on the stimuli-organism-response theory and the logistics service quality framework, integrates operational quality, resource quality, information quality, personal contact quality, customization quality, and customer satisfaction to study logistics service re-use intentions. The data were obtained from an online survey using a structured questionnaire given to those with experience in logistics service. Using partial least squares structural equation modeling on 810 respondents who were adult Chinese customers, this study discovered that operational, resource, information, personal contact, and customization qualities positively affect the satisfaction of logistics service customers, while customer satisfaction positively affects re-use intention. Moreover, the results of the mediation analysis revealed that customer satisfaction mediated the connection between the five components of LSQ and the re-use intention of logistics services. The originality of the study lies in its comprehensive examination of the direct and indirect effects of service quality dimensions on customer satisfaction and logistics service re-use intention in the context of logistics services. This study provides valuable insights into the importance of customer satisfaction in the logistics industry and highlights the need for logistics companies to prioritize customer satisfaction and improve their overall performance and competitiveness.

## Introduction

The internet has stimulated consumption, made shopping more convenient, and strengthened the development of the global economy. For sellers, logistics services are crucial for achieving competitive competence, especially in the presence of the internet and e-commerce [1]. As there is a significant interrelation between e-commerce and logistics, online purchases should be shipped through logistics, such as roads, railways, ships, and air, and logistics service quality and customer satisfaction should draw the attention of sellers [2]. Customers can buy goods online and choose logistics for their delivery. China has experienced the fastest growth in online shopping. The China Internet Network Information Center (CNNIC) data showed that as of June 2022, the number of internet users in China was 1.05 billion, an increase of 19.2 million since December 2021 [3]. Although the growth rate of the number of individuals who use the internet in China has recently decreased, the overall popularity of the internet in China is relatively high.

The logistics demand generated by online shopping is an important business component for logistics service providers (LSPs). Therefore, for LSP, it is necessary to research the connections among the elements of LSQ, customer satisfaction (CS), and logistics service re-use. LSPs must explore their capabilities to maintain stable and lasting customer relationships, guarantee, and meet customers’ continuously rising expectations regarding the quality of logistics services associated with online shopping [4]. For online stores, it is necessary to continuously enhance physical LSQ to support the special advantages of online shopping, such as convenience and low seeking costs.

Some LSQ frameworks are available in the literature; however, as noted by Juga et al. [5] and Vu et al. [4], most are geared toward developed countries. In Finland, Juga et al. [5] examined the effect of LSQ on CS and loyalty. However, China is a developing economy with a huge demand for logistics services. China’s logistics industry has experienced significant growth in recent years, with its market value surpassing 335 trillion yuan in 2021, which is double its previous value [6]. This rapid growth can be attributed to the flourishing domestic and cross-border e-commerce industries, which will see revenue exceeding 850 billion yuan in 2021 [6]. The COVID-19 pandemic and its lockdown have provided unique prospects for the rapid expansion of the e-commerce logistics sector. Unfortunately, comprehensive studies on the service quality (SQ) of the logistics industry in developing countries such as China are limited.

In existing studies, researchers have used both single and multiple dimensions to determine LSQ. Parasuraman et al. [7] established the significance of predators affecting SQ, which can affect customer satisfaction by shaping initial customer perceptions. Numerous researchers have expanded on Parasuraman’s work and developed LSQ scales and measurements. For instance, Mentzer et al. [8] expanded on the SERVQUAL framework in the US logistics sector to determine the key dimensions for assessing LSQ, including information quality (IQ), order release quantities, order accuracy, timeliness, ordering procedures, order condition, order discrepancy handling, personnel contact quality, and order quality. Sorkun et al. [9] studied LSQ and CS based on a single operational service quality dimension. Similarly, Uvet [10] tested the LSQ relationship with CS based on LSQ dimensions such as timeliness, order discrepancy handling, personal Contact Quality (PQ), operational information sharing, and order condition. Gupta et al. [11] studied several dimensions (such as operational, resource, information, customization-innovation quality, and personal contact) of LSQ with CS and customer loyalty. However, Uvet [10] and Gupta et al. [11] failed to portray the reuse intentions (RI) of logistics services and how LSQ acts as an antecedent. The repeated purchase of services ensures their value, which is essential for understanding the logistics service providers.

However, research on the mediation of CS between LSQ and RI is rare. Ahmed et al. [12] tested the mediation of CS within single-dimensional SQ and RI. A single dimension is very limited in representing all quality landscapes simultaneously. However, the mediating influence of satisfaction on the relationship between LSQ and RI is limited. Jain et al. [13] an exception, which researched the mediation of online shopping satisfaction between LSQ dimensions such as availability, timeliness, condition, and online repurchase intention. They experimented with dimensions that were limited to comprehensively understanding the logistics service quality. In particular, the mediation relationship between each logistics service quality dimension, such as operational, resource, information, personal contact, customization-innovation quality, and reuse intention, is nonexistent; thus, researchers have limited knowledge and access. Therefore, further research is required to establish indirect relationships and gain a comprehensive understanding of reuse intention dynamics.

To address these gaps, this study explores the connection between service quality (SQ) dimensions, CS, and RI for logistics services. It also explores the mediating effects of CS between SQ dimensions and RI. In view of the different cultures and environments in other countries and regions, this study starts from the Chinese market and examines the connection between the LSQ and RI of online shopping logistics in China, which has important theoretical and practical significance. The significance of this study lies in its contribution to the logistics literature by providing a comprehensive model that includes five dimensions of LSQ and their impact on CS and RI. This study offers CS as a significant mediator in the connection between LSQ dimensions and RI, which provides significant insights into the logistics literature. The empirical evidence provided by this study from a developing country (China) using a large sample size and structural equation modeling further enhances the credibility of the findings and provides a thorough understanding of the relationships. The policy implications of this research for logistics service providers can help improve customer satisfaction, increase service re-use intentions, and establish a loyal customer base, ultimately leading to competitive competence in the market. This paper includes four parts: the first part is the introduction, followed by the literature review, the model and research hypothesis, and finally the discussion. The following section presents a literature review of existing studies.

## Literature review

### Theoretical foundation

#### Stimulus-organism-response (SOR) model

The stimulus-organism-response (SOR) model, employed by Mehrabian and Russell in 1974, explains the role of environmental stimuli on emotions and future behaviors [14]. The SOR model is a theoretical framework used to explain the relationships between external stimuli, internal physiological and psychological responses, and behavioral outcomes. The SOR theory holds that stimulus, organism, and response, as the three stages of human behavior patterns, are closely related; stimulus is the external cause, the body is the intermediary link, and the response is the result of direct or indirect action of the stimulus and the body. According to this theory, the logistics operational quality (OQ), resource quality (RQ), IQ, PQ, and customization quality (CQ), which belong to the stimulus categories, may have a significant effect on CS in the response category. The dimensions of LSQ can be considered the external stimuli generated from the provided services. For example, when a customer interacts with a customer service representative who is polite and helpful, it creates a positive experience and perception of the service, which can influence the customer’s likelihood of reusing the service. Similarly, if a logistics company provides timely and accurate information about the status of a customer’s delivery, it can create a positive perception of the service quality and improve the likelihood of customer satisfaction and reuse intention. Besides, the study of Tian et al. [15] and Nunthiphatprueksa [16] used service quality as a stimulus under SOR theory in their organic tea and tourism research respectively. Based on the SOR theory, this study regarded the elements related to the SQ of online shopping logistics as the stimulus variable (S), CS as the organism variable (O), and logistics service RI as the reaction variable (R), and explored the mechanism of the influence of logistics service RI. To theoretically enrich customers’ willingness to re-use logistics services in the online shopping environment and provide theoretical guidance to seize the huge potential market of e-commerce logistics.

### Logistics service quality

Logistics service quality refers to the extent to which logistics services meet or exceed the expectations of customers. Recently, scholars studied LSQ extensively. The term “logistics service quality” was first introduced and explored by Mentzer et al. [17]. They argued that customers’ perceptions of LSQ include not only physical distribution services but also other aspects that are of great importance to them. As many studies have verified, service performance is an important driver element in the logistics domain that creates value and helps obtain competitive advantages [8, 18, 19]. In most studies, LSQ is examined and estimated in various dimensions. The LSQ framework proposed by Mentzer et al. [8] includes nine service quality dimensions: PQ, IQ, ordering procedures, order quality, ordering release quantities, order discrepancy handling, order accuracy, timeliness, and order conditions. Furthermore, Mentzer et al. [19] explored logistics services in three dimensions: condition, timelessness, and availability. The viewpoint of Mentzer et al. [8] was also accepted and explored by Bienstock et al. [20] in their study on the SQ of physical distribution as a double-order construct consisting of timeliness, availability, and conditions. Previous findings also revealed that LSQ has a two-dimensional variable—operational and relational performance—in line with Stank et al. [18] and Daugherty et al. [21]. Rafele [22] proposed a framework for estimating logistics service performance, including logistics quality, in three dimensions: fulfillment methods, tangible components, and information actions. In line with Saura et al. [23], LSQ is mainly determined by empathy and reliability. Bouzaabia [24] studied 12 items of the LSQ from Mentzer et al. [19] and eliminated three items; the remaining nine items were structured by accounting for two dimensions: operational LSQ and relational LSQ. Gil-Saura et al. [25] proposed personal quality, information quality, order quality, punctuality, and order form [25]. In line with Gaudenzi [26], the key variables that characterize the wide LSQ concept have been discussed in seven dimensions: IQ, PQ, ordering procedures, order accuracy, order condition, timeliness, and order discrepancy handling. The perspectives of Gil-Saura et al. [25], Gaudenzi [26], and others were partially adopted in this study. Hafez et al. [27] explored six dimensions of LSQ such as information quality, product quality, product condition, delivery services, reverse logistics, customer services, while Revindran et al. [28] identified four dimension like product availability, Timeliness, product condition and reverse logistics. Choi et al., [29] proposed five dimensions like quality of delivery, quality of information, price of delivery quality of order, and customer service. Likewise, Rajendran et al. [30] suggested five dimensions such as Delivery services, reverse logistics, product quality, customer service and service recovery.

**Fig 1:**
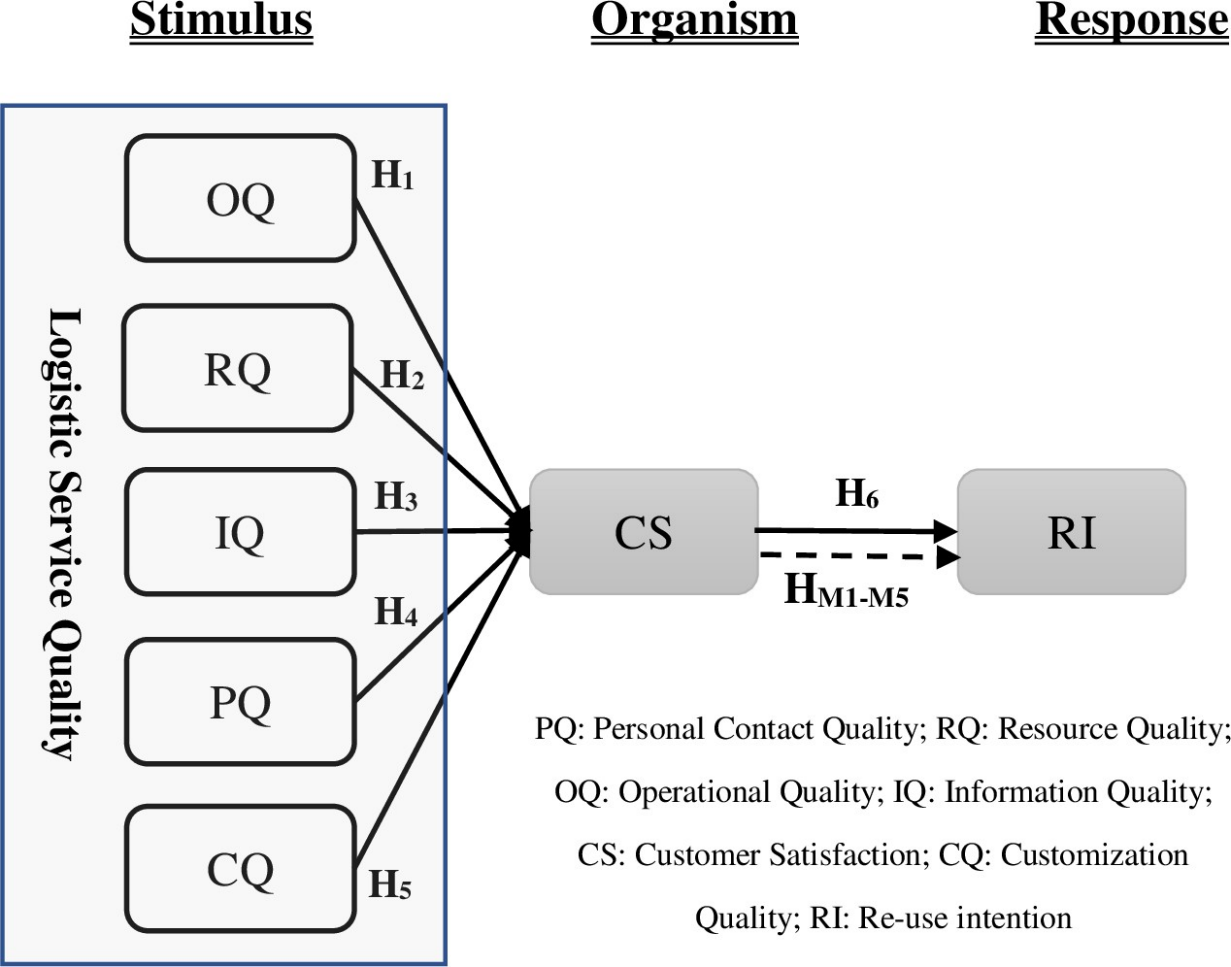
Conceptual framework of re-use intention.

Owing to the rapid increase in B2C and other online commerce, a limitation due to insufficient LSQ is outstanding, whereby examining electronic LSQ is crucial in a B2C commerce environment [31]. Concerning electronic LSQ (e-LSQ), Rao et al. [32] introduced a measurement framework for e-LSQ in the B2C environment, which included order tracking, on-time delivery, item availability, and shipping options. However, in present research logistics service quality refers to the overall evaluation of the quality of logistics services provided by a company, which comprises five dimensions: personal contact quality, resource quality, operational quality, information quality, and customization quality (Fig 1). These dimensions collectively describe the attributes of logistics services that are crucial in meeting customers’ needs and expectations, such as the quality of communication, resources, operations, information, and the degree to which services are customized to meet individual customer requirements.

### Customer satisfaction

CS is the emotional response of customers when they perceive a distinction between their past expectations and the real performance of a product or service that they have used [33]. It is considered an overall judgment of performance [34, 35]. CS in logistics industry context indicates that the customers’ delightfulness, well matching services with expectations, positive or better feelings towards LSP for choosing them. Many studies have shown that improvements in LSQ contribute to CS [21, 36], and ensuring CS with physical delivery services is a crucial objective for LSP. Extensive research has been conducted on CS in relation to logistics services [3, 19, 37]. Previous studies have considered CS to be a result of LSQ and investigated how LSQ affects CS [19, 38]. Stank et al. [18] found that relational performance has a considerable effect on CS, whereas operational performance has no such effect. Furthermore, optimizing LSQ can lead to improved CS [39]. As CS and SQ are interconnected concepts that are extensively studied in the LSQ literature, it is essential to identify which factors of LSQ have a significant impact on CS.

Numerous studies have confirmed the importance of LSQ for achieving CS. Rafiq and Jaafar [38] described the link between customer perception of LSQ and its impact on CS, considering functional measures, such as PQ, IQ, and ordering procedures. Most studies have verified the relationship between LSQ and CS in a B2B environment between industrial purchasers and vendors [17, 19, 40, 41]. Murfield et al. [31] studied the effect of LSQ on CS and customer loyalty in an omnichannel commerce environment and concluded that three components of LSQ—timeless, availability, and condition—have positive associations with the CS of logistics services. Studies in a B2C environment have confirmed a positive correlation between LSQ and CS in online retail [42]. Rao et al. [32] also found that item availability and online delivery are critical elements of electronic physical distribution service quality. However, most studies have focused on customer purchase satisfaction rather than CS with logistics services.

### Re-use intention

The RI is an important method for measuring customer behavior in marketing studies. The business is committed to strengthening customer loyalty by increasing repurchases and re-use, preventing customer loss, and increasing the retention ratio by maintaining high customer loyalty. In the past few years, the intention of reusing has received a significant stream of attention from researchers. Furthermore, it has been extensively used in studies on customer loyalty. Different studies concerning different industries, such as food delivery [43], mobile bookstore applications [44], e-commerce [45], and smart tourism [46], have indicated that customer satisfaction affects re-use intention. Jen et al. [47] and de Oña et al. [48] specified that excellent CS is directly connected to customer RI. Furthermore, as Chou and Kim [49] and Jen et al. [47] concluded, customer satisfaction is an interlink between service quality and re-use intention.

For logistics service providers, the re-use intention of customers is very important from a long-standing perspective, which ensures that both receivers and providers of logistics services have a favorable relationship [50]. In line with the existing research, reserving existing customers in other domains is considerably more meaningful than achieving new customers. The intention to re-use a logistics service can be defined as the customer’s belief and willingness to make up a product and express it as a specific future behavior i.e., willing to use or recommend other about the logistics services or service providers in future when needed to use. To some extent, logistics service re-use intention is critical to the success of sellers, not only from the viewpoint of the sellers but also from that of the buyers. Through an online investigation of online sellers, Ngah et al. [45] concluded that satisfaction significantly relates to the re-use intention of certain third-party logistics services and that the satisfaction index was a mediator between reliability and RI. At present, there is limited literature on the logistics service re-use intentions from the perspective of online shopping customers.

### Hypotheses development

Following Mentzer et al. [8], nine constructs have been proposed, including information quality and ordering procedures. CS and quality of service are related concepts that have been widely studied by various scholars. Mentzer et al. [19] insisted that CS was a consequence of the LSQ, time of delivery, and settlement of orders’ puzzles and problems, significantly reducing satisfaction. Studies have stated that operational and relational performances related to logistics services have significant and positive relationships with CS [21]. According to Ramanathan [51], the primary cause of customer dissatisfaction stems from either late or non-delivery of products, inaccurate orders, or damaged products. Yeo et al. [52] and Gupta et al. [11] showed a significant link between RQ and CS logistics. Based on this reasoning, the following hypotheses (H_1_, H_2_) are proposed:

**H**_**1**_**:** *OQ positively influence CS*.
**H**_**2**_**:** *RQ positively influences CS*.

There is insufficient personal contact between logistics service providers and buyers; therefore, information delivered by logistics service providers through the web or apps is especially important. When logistics service providers provide high-quality information to their customers, they enhance the customers’ overall experience, leading to greater CS. For example, when customers receive authentic and upgraded information about the status of their shipments, they feel more informed and in control of the delivery process [10, 53]. This can help reduce uncertainty and anxiety, which, in turn, leads to greater satisfaction with logistics services.

Similarly, when logistics service providers provide complete information about the range of services they offer, they help customers make informed decisions about which services to use [3]. This can lead to greater customer satisfaction as they can select the services that best meet their needs. Additionally, relevant information tailored to customer-specific requirements can lead to greater satisfaction [10]. For instance, providing customers with information about the expected delivery time, delivery cost, and options for rerouting or rescheduling their shipments can help them meet their individual needs and preferences, ultimately leading to a more satisfying customer experience. Thus, high-quality information provided by logistics service providers can lead to greater customer satisfaction by reducing uncertainty, helping customers make informed decisions, and meeting their individual needs and preferences. Thus, we propose the following hypothesis:

**H**_**3**_**:** *IQ positively influences the CS*.

Bouzaabia [24] compared the perceptions of retail logistics service quality among Romanian and Tunisian customers and found that relational logistics service quality was the most vital interpreter of satisfaction. According to Uvet’s [9] findings on the correlation between consumers and employees in their awareness of SQ in logistics, it was determined that CS with logistics services is also influenced by the behavior and attentiveness of LSP staff. And they specifically found that PQ has a significant impact on CS. Similarly, Gupta et al. [11] revealed that personal contact quality affects CS positively in the case of logistics services. Based on these arguments, this study hypothesizes the following:

**H**_**4**_**:** *PQ positively influences CS*.

Coelho and Henseler [54] declared that customization strengthens service quality, perceived customer trust, CS, and customer loyalty toward service providers. For logistics service providers, customer-specific or customized requirements are important for satisfaction and establishing a close relationship. In line with Hu [55], customized logistics services are a predictor of CS. Gupta et al. [11] discovered that the customization and innovation have a positive effect on CS for logistics services. Thus, the following hypothesis was derived:

**H**_**5**_**:** *CQ positively affects CS with logistics service*.

In line with Oliver [33], CS impacts customer attitudes and leads to a significant positive effect on potential behaviors in the future, such as RI. The customer intention to re-purchase (re-use) products (services) covers specific and cautious attitudes because of experiences, particularly when customers concern a specific product or service [35]. Positive attitudes originate from satisfaction with products or services, and later impact intentions [56–58]. According to Wang et al. [59] and Alalwan [60], CS has a significant impact on RI in service industries, such as mobile food ordering online [60] and urban rail transit [59]. In the Malaysian context, Ngah et al. [45] found that satisfaction positively influences the intention to reuse third-party logistics services. Especially for online shopping linked to physical distribution, customers are concerned not only with online apps or websites but also with logistics services to complete purchases. Based on the above discussion, to test the effect of CS on the RI of logistics services, the following hypothesis was proposed:

**H**_**6**_**:** *CS positively affects logistics service RI*.

According to the SOR model, the two dimensions of customer emotion and customer cognition are included in the organism variables, whereas customer satisfaction is included in customer emotion. As a response variable, research on customer behaviors in the theoretical area generally revolves around two paths: first, as per the Mehurabian-Russell (M-R) framework, stressing the intermediary role of emotion in environmental stimulus and customer response [61]; second, using perceived service quality as the intermediary variable, emphasizing that customers generate self-cognition through external stimulation, which then affects purchase intention [62]. In Zajonc’s [63] opinion, the two paths should be considered together; that is, both the cognitive and emotional responses should be studied.

Based on the M-R model, emotions play a mediating role in environmental stimuli and customer responses. This has been previously verified by several researchers. In line with Dabholkar et al. [64], elements relating to the quality of service are better designed as antecedents than their components, and CS is a significant mediator between SQ and behavioral intentions, such as the intention to re-use or re-purchase. In line with Yilmaz et al. [65] and Wang [59], positive CS created by the quality of service provided by the metro operator led to re-use intention. In line with Preacher et al. [66], a mediating effect occurs when a predicting variable indirectly affects a dependent variable through at least one intervening variable or mediator. Based on the previous literature reviews, this study concludes that customer satisfaction as a mediating variable significantly affects customers’ willingness to re-use logistics services. Thus, the following hypotheses were proposed:

**H**_**M1**_**:** *CS is a significant mediator within the OQ and logistics service RI relationship*.
**H**_**M2**_**:** *CS is a significant mediator within RQ and logistics service RI relationship*.
**H**_**M3**_**:** *CS is a significant mediator within IQ and logistics service RI relationship*.
**H**_**M4**_**:** *CS is a significant mediator within PQ and logistics service RI relationship*.
**H**_**M5**_**:** *CS is a significant mediator within CQ and logistics service RI relationship*.

All association hypothesized above, presented in Fig 1 below.

## Research methodology

In our study, quantitative analysis was used to verify the relationship among logistics SQ, logistics CS, and logistics service RI. We used a cross-sectional survey design, posting an online self-administered questionnaire to the Sojump website, and circulating the questionnaire through social media, such as WeChat and QQ, for convenience sampling.

### Population and sample

The targeted respondents of this study were Chinese customers older than 18 years, representing approximately 65% of the Chinese population. The minimum sample size in this study was calculated using G*Power software. With an effect size (f^2^) of 0.15, a power of 0.95, and six exogenous variables, the minimum sample size required was 146 [67]. A sample size of 810, obtained from the distribution of 832 questionnaires, resulting in a response rate of 95.08 percent, was deemed sufficient for this study. Consequently, the subsequent investigation was considered valid. Our continuous effort to remind the participants to complete the questionnaire was the reason for the high response rates. The human research ethics committee of Nantong Institute of Technology approved this study (Reference number: BS-NIT-2023-0402). This study has been performed in accordance with the Declaration of Helsinki. This study collected written informed consent from all respondents. This study collected and reported no identifiable information. The information was gathered between November 15 and December 22, 2022.

### Survey instrument

The questionnaire comprised two parts. The first was the demographic profile of the participants, such as educational level, age, average monthly income, gender, marital status, employment status, online shopping expense cost, and location. In addition, the interviewees were required to reply about the frequency with which they purchased products online and used logistics services each month. The second section focused on the exogenous and endogenous constructs of logistics services, including OQ, RQ, IQ, PQ, CQ, CS, and RI. All 36 measurement items of the above constructs were rearranged in accordance with previous studies. Five items of operational quality were adopted from Bouzaabia et al. [24]; Juga et al. [5]; and Gronroos [68]. Following the research of Gupta et al. [11]; Yeo et al. [52]; Thai [69], five items of resource quality were chosen. The five items of information quality were extracted from the study of Thai [70]; Huang et al. [71]; Saura et al. [23]; and Rafiq and Jaafar [38]. The study picked 5 items for personal contract quality from the studies of Bienstock et al. [20]; Saura et al. [23]; Rafiq and Jaafar [38]; and Mentzer et al. [19], while the five items of customization quality were adapted from the study of Vu et al. [4]; Hu et al. [55]; and Coelho and Henseler [54]. The study of Kwak et al. [72] was followed for the items (five) of reuse intention construct, whereas the study of Ali et al. [37]; Vu et al. [4]; Klaus and Maklan [73]; Dagger et al. [74]; Mentzer et al. [19] were consulted for the six items of customer satisfaction. The respondents were suggested at the beginning of the questionnaire to fill in the questionnaire according to their latest or most impressive service experience from logistics providers. To measure all indicators of the variables, we used a seven-point Likert scale ranging from 1 (strongly disagree) to 7 (strongly agree). Full information on the questionnaire used to measure all variables is presented in ***Survey Questionnaire in S1 File***.” The questionnaire was developed in English and later translated into Chinese to inquire about adult Chinese customers. To ensure that the responses were not distorted or misunderstood, professional translators were invited to assist in the translation work.

### Data collection method

The online questionnaire was uploaded at the link “https://www.wjx.cn/vm/eBmy8hA.aspx#.”. A convenience sampling strategy was adopted to obtain adequate responses to the questionnaire via both WeChat and QQ software from November 12, 2022, to November 23, 2022, and a qualifying question was used to filter the potential respondents. Consequently, 832 responses were successfully obtained and 21 (2.52%) respondents chose “never use the service from the logistics service providers,” which was set aside. Consequently, 810 valid samples were retained for subsequent analysis. Informed consent was obtained from all participants using a questionnaire for ethical approval. To ensure the accuracy and efficiency of data collection during the survey, we conducted a pilot test. We closely monitored the distribution and completion of questionnaires to ensure their validity and organization. Our pre-test sample consisted of 35 participants, from whom we collected the completed questionnaires. The results from the pre-test confirmed the initial validity and reliability of the survey items. Complete data presented in **Dataset in S2 File.**

### Common method bias

In this study, a self-administered questionnaire was adopted for data collection, and a structural model was used to assess the hypothesis. As a result, common method bias (CMB) could occur. Hence, both procedural and statistical remedies were used to avoid CMB [75]. In case of procedural measures, terminology definitions were clearly described and provided clear and concise instructions for respondents to follow when completing surveys in the questionnaire. This is helpful to reduce confusion and errors that can lead to bias in the data. Also, we told the people who answered them that there were no absolute right or wrong response and that their answers would be assessed anonymously. This can help to reduce the potential for social desirability bias and CMB that can arise when respondents feel pressured to provide certain answers. Besides, we randomized the order of questions to reduce the potential for order effects or other biases that can arise when questions are presented in a certain order. Regarding the statistical measures, first, the study conducted a Harman’s Single factor test and revealed that a single factor explained 39.44% of the overall variance, which is less than 50%, and CMB did not exist [76]. Second, the CMB was evaluated using Kock’s [77] full collinearity test. All the latent variables in this study were regressed on a commonly created variable. The results of the full collinearity test and variance-inflated factor (VIF) values for OQ (1.378), RQ (1.727), IQ (1.821), PQ (1.854), CQ (1.888), CS (2.065), and RI (2.634) were all below 3.3, which are shown in Table 1, suggesting that the model did not meet the problem of CMB [77].

**Table 1:** Full collinearity test.

	OQ	RQ	IQ	PQ	CQ	CS	RI
Variance Inflation Factors	1.378	1.727	1.821	1.854	1.888	2.065	2.634

Note: PQ: Personal Contact Quality; RQ: Resource Quality; OQ: Operational Quality; IQ: Information Quality; CS: Customer Satisfaction; CQ: Customization Quality; RI: Re-use intention

### Multivariate normality

Conducting proper data analysis by checking for multivariate normality is vital. In this study, multivariate normality was estimated using the Web Power online tool [78]. Results of the multivariate normality test showed that the *p*-values for Mardia’s multivariate skewness and kurtosis were below 0.05, which certified the issue of non-normality [79].

### Data analysis method

The analysis was conducted in three stages. First, we quantified the validity and reliability of the measurement model. In the next phase, structural equation modeling (SEM) was used to elaborate the connection between the predictor and latent variables, including mediation and moderation effects. It is widely accepted that SEM provides better estimates than regression when executing mediation and moderation [80]. Therefore, this study applied SEM, specifically partial least squares-based SEM (PLS-SEM), using Smart-PLS 4.0. PLS-SEM was considered the best choice because of its effectiveness in evaluating complex frameworks that involve moderation effects [81]. Finally, to ensure the robustness of the results, a necessary condition analysis (NCA) using the bottleneck analysis approach was performed to achieve a comprehensive understanding of the relationships among the constructs. Necessity logic is critical for achieving a specific outcome, and its absence guarantees failure [82]. NCA is a statistical analysis approach that identifies single necessary causes or independent variables to achieve a target outcome [82]. Combining PLS-SEM and NCA has been suggested to further theorize and validate the theory (Richter et al., 2020). This technique is used as a complement to the existing conventional techniques i.e., here PLS-SEM identifies the factors that provide the best possible outcome while, NCA explore for necessity logic, and provides us necessary conditions to be meet for a particular outcome. This combined use leverage researchers to improve the accuracy and efficiency of the PLS analysis, as it reduces the number of variables that need to be included in the model.

## Findings and discussion

### Demographic characteristics of respondents

This study obtained valid results from 810 respondents. Table 2 presents the participants’ demographic profiles. Especially, the respondents were approximately equally split between males (49.8%) and females (50.2%). Besides that, 35.9% of participants were aged between 36 and 45 years, followed by participants aged 26–35 years (25.9%), 18–25 years (18.8%), 46–55 years (13.5%), and 56–65 years (4.7%). The remaining 1.2% of respondents were over 65 years old. Of the respondents, 33.5% were from East China, followed by North China (17.5%), Central China (15.7%), south China (11.9%), South China (11.9%), Northwest China (5.4%), South China (2.7%), and others (1.5%). In terms of monthly shopping frequency, 38.4% reported shopping 1–2 times per month, followed by 37.5% shopping 3–4 times per month, 13.5% shopping 6–10 times per month, and 9.3% shopping more than ten times per month, while only 1.4% reported rare shopping. The participants who used the logistics service 3–5 times were 42.7%, followed by 1–2 times (36.4%), and 6–10 times (12.1%). Of the respondents, 79.2% worked, and the remainder (20.9%) did not work. Of the respondents, 66.1% had a monthly income below RMB 4500, while the remaining 33.9% had an income above this threshold. Similarly, 85.8% of participants spent less than RMB 4500, while the remaining 14.2% spent more than that.

**Table 2:** Demographic characteristics (N = 810).

	n	%		n	%
*Gender*			*Logistics Service Use Frequency*		
Male	403	49.8	1–2 times per month	295	36.4
Female	407	50.2	3–5 times per month	346	42.7
Total	810	100.0	6–10 times per month	98	12.1
			Above 10 times per month	71	8.8
*Age Group*			Total	810	100.0
18–25 years	152	18.8			
26–35 years	210	25.9	*Employment Status*		
36–45 years	291	35.9	Employed Full Time	269	33.2
46–55 years	109	13.5	Employed Part Time	264	32.6
56–65 years	38	4.7	Self-employed	108	13.3
More than 65 years	10	1.2	Student	126	15.6
Total	810	100.0	Unemployed	34	4.2
			Retired	9	1.1
*Monthly Shopping Times (Per Month)*			Total	810	100.0
Rarely	11	1.4			
1–2 times	311	38.4	*Monthly Income*		
3–5 times	304	37.5	Less than RMB1500	136	16.8
6–10 times	109	13.5	RMB1500-RMB3000	181	22.3
Above 10 times	75	9.3	RMB3001-RMB4500	219	27.0
Total	810	100.0	RMB4501-RMB6000	84	10.4
			RMB6001-RMB7500	50	6.2
*Location*			More than RMB 7500	140	17.3
Northeast China	96	11.9	Total	810	100.0
North China	142	17.5			
East China	271	33.5	*Monthly Online Shopping Expenses*		
Central China	127	15.7	Less than RMB1500	247	30.5
South China	96	11.9	RMB1500-RMB3000	276	34.1
Northwest China	44	5.4	RMB3001-RMB4500	172	21.2
Southwest China	22	2.7	RMB4501-RMB6000	74	9.1
Others	12	1.5	RMB6001-RMB7500	29	3.6
Total	810	100.0	More than RMB 7500	12	1.5
			Total	810	100.0

**Note:** 1 USD = 7.15 RMB

### Reliability and validity

To confirm the construct reliability utilized in this study, internal consistency reliability was evaluated by computing both Cronbach’s alpha and composite reliability (rho_a and rho_c) (as presented in Table 3). According to Hair et al. [81], the composite reliability values (rho_c) for all constructs met the recommended range of 0.70–0.90. Additionally, the composite reliability values (rho_a) for all variables were within the lower and upper bounds of Cronbach’s alpha and composite reliability (rho_c), thus confirming a high level of internal consistency among the constructs. In addition, the average variance extracted (AVE) for all items for each variable was higher than 0.50, establishing adequate convergent validity to affirm the unidimensionality of each variable [83]. Therefore, we found sufficient reliability in the constructs.

**Table 3:** Reliability and validity.

Variables	No. Items	Mean	Standard Deviation	Cronbach’s Alpha	Composite reliability (rho_a)	Composite reliability (rho_c)	Average Variance Extracted	Variance Inflation Factors
OQ	5	3.878	1.861	0.963	0.964	0.971	0.870	1.202
RQ	5	5.307	1.282	0.922	0.923	0.941	0.763	1.565
IQ	5	5.266	1.251	0.925	0.926	0.943	0.768	1.623
PQ	5	5.333	1.178	0.904	0.905	0.929	0.723	1.662
CQ	5	5.585	1.114	0.885	0.888	0.915	0.684	1.658
CS	6	5.257	1.234	0.905	0.905	0.926	0.677	1.000
RI	5	5.695	1.141	0.902	0.904	0.928	0.719	-

Note: Personal Contact Quality (PQ); Resource Quality (RQ); Operational Quality (OQ); Information Quality (IQ); Customer Satisfaction (CS); Customization Quality (CQ); Re-use Intention (RI)

Source: Author’s data analysis.

**Fig 2:**
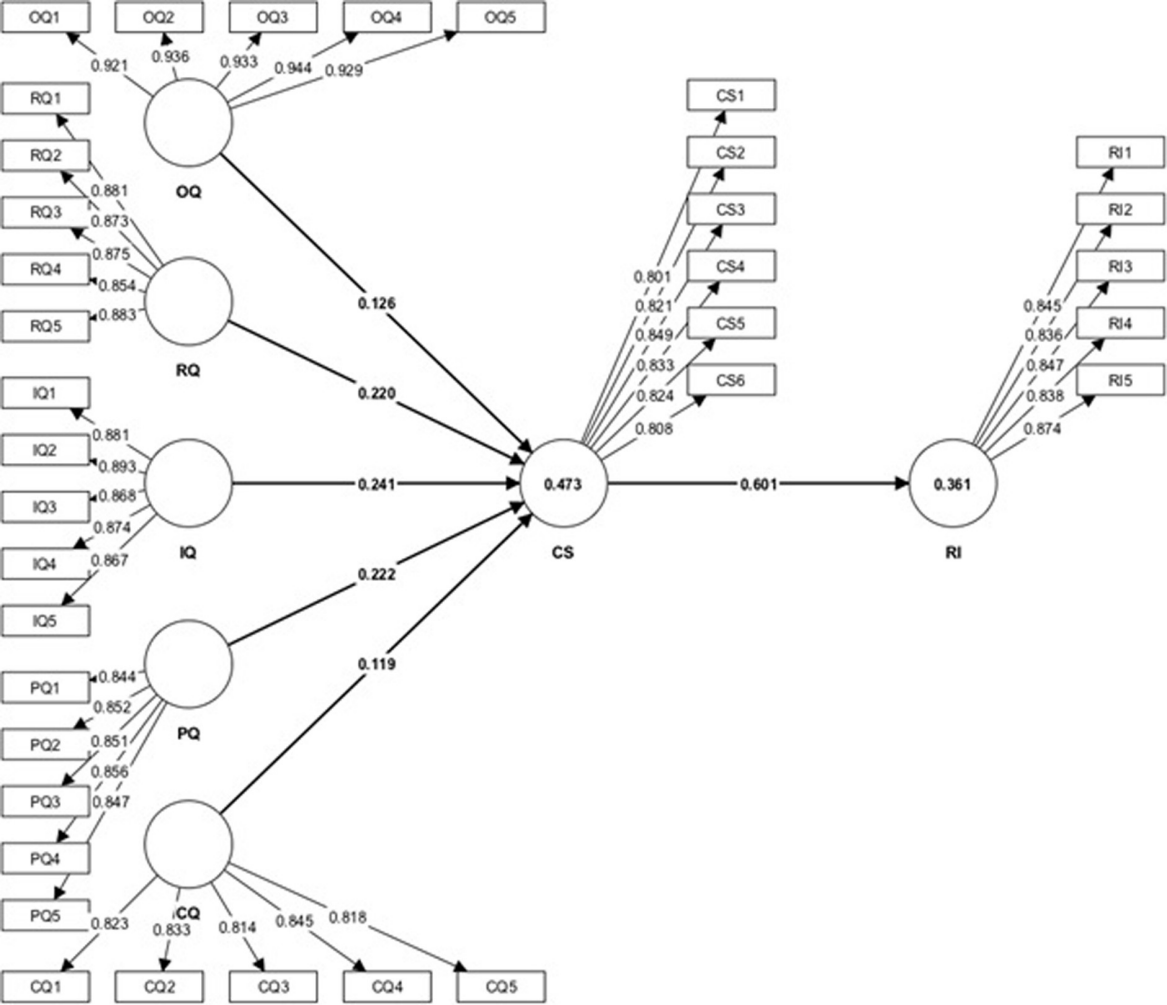
Measurement model.

Once reliability was confirmed, the discriminant validity of the model was assessed by applying both Fornell and Lacker’s criteria and the heterotrait–monotrait ratio (see Dataset in S2 File. Loading, Cross-Loading, and the Fornell-Larcker criterion; and Fig 2). The discriminant validity of the framework was evaluated using Fornell and Larcker’s criterion, and the results satisfied this criterion, as presented in Table 4. According to the analysis outcome, the model displays discriminant validity, which is evident from the higher value of the square root of the AVE in the diagonal compared to the off-diagonal constructs [84]. This finding suggests that the construct measures in the model are more closely related to their respective variables than to other variables, indicating a good level of discriminant validity. According to Henseler et al. [85], an optimum heterotrait-monotrait ratio of correlations (HTMT) below 0.90 indicates high levels of discriminant validity (Table 4). Although the value below 0.85 is sufficient, we used higher threshold value so that it ensures that the constructs are sufficiently distinct and do not overlap in terms of the underlying constructs they are measuring. Also, by using a higher threshold, researchers can be more confident that their measures are truly distinct and can provide more accurate and reliable results. In this study, all values in the HTMT matrix were below this threshold, confirming a greater extent of discriminant validity.

**Table 4:** Heterotrait-monotrait ratio (HTMT) matrix.

	CQ	CS	IQ	OQ	PQ	RI	RQ
CQ	-						
CS	0.526	-					
IQ	0.515	0.607	-				
OQ	0.141	0.378	0.368	-			
PQ	0.620	0.592	0.534	0.258	-		
RI	0.715	0.662	0.602	0.066	0.656	-	
RQ	0.515	0.587	0.528	0.348	0.503	0.583	-

Note: Personal Contact Quality (PQ); Resource Quality (RQ); Operational Quality (OQ); Information Quality (IQ); Customer Satisfaction (CS); Customization Quality (CQ); Re-use Intention (RI)

To determine multicollinearity, this study quantified the variance inflation factors (VIF) for all measurement items. All VIF scores were below the recommended cut-off value of 5.0, indicating the absence of multicollinearity. Therefore, the framework used in this study does not suffer from multicollinearity issues. Finally, the findings suggest that the model used in the study is reliable and valid and that the factors are independent of each other.

### Hypothesis testing

The determination coefficient *R*^*2*^ showed the explanatory power of the prediction model equations in explaining the endogenous constructs. According to Cohen’s [86] recommendations, an endogenous construct’s R^2^ value ranging from 0.02 to 0.12 is generally deemed weak, while a value between 0.13 and 0.25 is regarded as medium, and a value of 0.26 or higher is considered substantial. As shown in Fig 2, the proposed model showed substantial explanatory power for CS (0.473) and logistics RI (0.361).

For the direct hypothesis (Fig 2 and Table 5), OQ (beta = 0.126, *p* < 0.01), RQ (beta = 0.220, *p* < 0.01), IQ (beta = 0.241, *p-value* < 0.01), PQ (beta = 0.222, *p-value* < 0.01), and CQ (beta = 0.119, *p* < 0.01) have significant relationships with CS for logistics services. CS (beta = 0.601, *p*<0.01) is significantly connected to logistics service RI. Notably, the results for H_1_, H_2_, H_3_, H_4_, and H_5_ indicated that R^2^ was 0.473 and OQ, RQ, IQ, PQ, and CQ explained 47.3% of the variance in the CS. Meanwhile, the results of H_6_ also revealed that CS explained 36.1% of the variance in logistics service RI, with beta = 0.601, *p* < 0.01, and R^2^ (0.361). Therefore, H_1_, H_2_, H_3_, H_4_, H_5_, and H_6_, which were proposed to test the direct effect, were all supported at the 1% significance level.

**Table 5:** Hypothesis testing.

Hypothesis		Beta	CI Min	CI Max	*t* Value	*p* Value	*R* ^*2*^	Decision
H_1_	OQ → CS	0.126	0.078	0.174	4.249	0.000	0.473	Supported
H_2_	RQ → CS	0.220	0.150	0.291	5.121	0.000		Supported
H_3_	IQ → CS	0.241	0.169	0.313	5.540	0.000		Supported
H_4_	PQ → CS	0.222	0.147	0.296	4.887	0.000		Supported
H_5_	CQ → CS	0.119	0.054	0.185	2.940	0.002		Supported
H_6_	CS → RI	0.601	0.543	0.657	17.404	0.000	0.361	Supported
H_M1_	OQ → CS → RI	0.076	0.048	0.102	4.592	0.000		Supported
H_M2_	RQ → CS → RI	0.133	0.088	0.180	4.762	0.000		Supported
H_M3_	IQ → CS → RI	0.145	0.099	0.191	5.186	0.000		Supported
H_M4_	PQ → CS → RI	0.133	0.087	0.182	4.581	0.000		Supported
H_M5_	CQ → CS → RI	0.072	0.031	0.115	2.790	0.003		Supported

Note: Personal Contact Quality (PQ); Resource Quality (RQ); Operational Quality (OQ); Information Quality (IQ); Customer Satisfaction (CS); Customization Quality (CQ); Re-use Intention (RI)

The study also explored the mediation hypothesis by applying bootstrapping to the indirect influence, in line with Preacher et al. (2008). The result (Table 5) reveals that CS contributed a significant and positive mediating role for the connection among OQ → CS → RI (β-value = 0.076, *t-value* = 4.592, *p-value*<0.01, CI Min = 0.543, CI Max = 0.657), RQ → CS → RI (β-value = 0.133, *t-value* = 4.762, *p-value*<0.01, CI Min = 0.088, CI Max = 0.180), IQ → CS → RI (β-value = 0.145, *t-value* = 5.186, *p-value*<0.01, CI Min = 0.099, CI Max = 0.191), PQ → CS → RI (β-value = 0.133, *t-value* = 4.581, *p-value*<0.01, CI Min = 0.087, CI Max = 0.182), and CQ → CS → RI (β-value = 0.072, *t-value* = 2.790, *p-value*<0.01, CI Min = 0.031, CI Max = 0.115). As a result, at the 1% significance level, the hypotheses H_M1_, H_M2_, H_M3_, H_M4_, and H_M5_ that CS mediates the relationships of OQ, RQ, IQ, PQ, and CQ with RI were supported.

#### Necessary condition analysis

**Fig 3:**
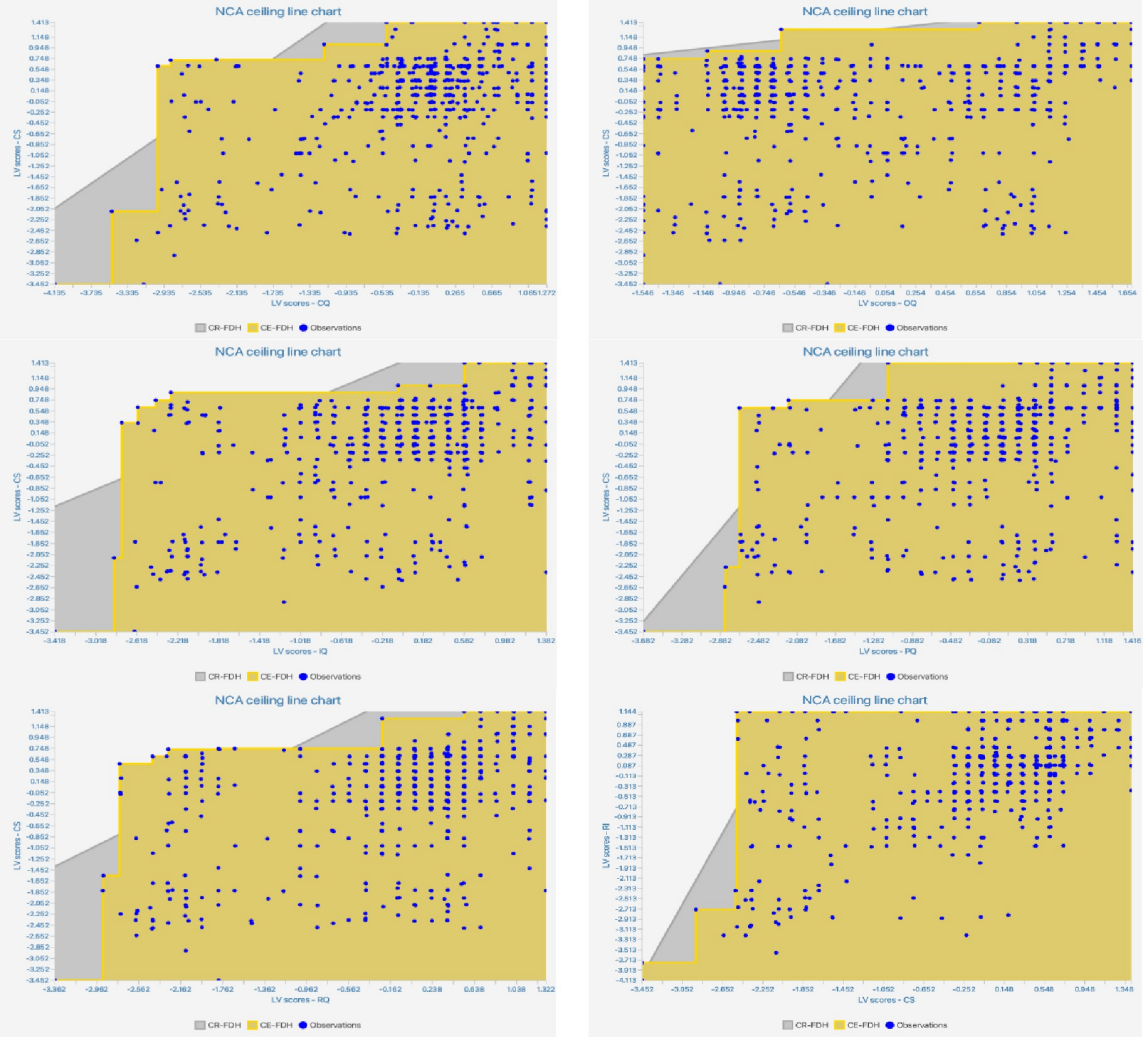
NCA charts.

NCA can identify whether the existence of a specific factor is a necessary condition for the emergence of a certain result, and it can also analyze the bottleneck extent of the necessary condition [82]. Fig 3 reveals the scatter plots for all connections, and Table 6 reveals the NCA effect size *d* value, which is a “quantitative reflection of the magnitude of some phenomenon that is used to address a question of interest” [87]. For general studies, 0<d<0.1 is a “small effect,” 0.1<d<0.3 is a “medium effect,” 0.3<d<0.5 is a “large effect,” and d≥0.5 is a “very large effect.” [82]. In this study (Table 6), the effect sizes were between 0.1 and 0.3, except for information quality, which had the lowest effect size (0.045). The effect sizes were deemed to be both theoretically and practically meaningful, as concluded by Van et al. [88]. As directed by Kelley & Preacher [87], with the effect size of 0.1 as the standard, X “information quality” is not necessary to Y “customer satisfaction.” The NCA’s results manifest that OQ, RQ, PQ, and CQ are meaningful (d≥0.1) and significant (p<0.05) necessary conditions for CS; CS is logical and significant necessary conditions for logistics service RI.

**Table 6:** NCA effect size.

	Customer Satisfaction		Re-use Intention	
	Original effect size	Permutation p-value	Original effect size	Permutation p-value
Operational Quality	0.243	0.000		
Resource Quality	0.211	0.000		
Information Quality	0.045	0.000		
Personal Contact Quality	0.234	0.000		
Customization Quality	0.202	0.000		
Customer Satisfaction			0.164	0.000

Note: Personal Contact Quality (PQ); Resource Quality (RQ); Operational Quality (OQ); Information Quality (IQ); Customer Satisfaction (CS); Customization Quality (CQ); Re-use Intention (RI)

Table 7 reports the bottleneck-level analysis results of the NCA. As can be seen, to achieve 50% CS, a 0.494% of RQ, 0.37% of IQ, 1.111% of PQ, and 1.111% of CQ are required, while there is no bottleneck level in OQ; to achieve 90% CS, all 5 components of LSQ are bottlenecks for CS. The study also investigated whether customer satisfaction is a necessary condition for re-use intentions. Table 7 highlights this while considering the relationship between CS and RI, to achieve 30% re-use intention, a 1.111% CS is required; to achieve 40% or above levels of re-use intention, a 1.235% CS is required.

**Table 7:** Bottleneck percentages.

Percentage	Customer Satisfaction					Re-use Intention
	OQ	RQ	IQ	PQ	CQ	CS
0.00%	NN	0.247	0.247	0.37	0.247	NN
10.00%	NN	0.247	0.247	0.37	0.247	0.37
20.00%	NN	0.247	0.247	0.988	0.247	0.37
30.00%	NN	0.247	0.37	1.111	1.111	1.111
40.00%	NN	0.494	0.37	1.111	1.111	1.235
50.00%	NN	0.494	0.37	1.111	1.111	1.235
60.00%	NN	0.494	0.37	1.111	1.111	1.235
70.00%	NN	0.494	0.37	1.111	1.111	1.235
80.00%	NN	0.494	1.235	1.111	1.111	1.235
90.00%	38.889	25.432	37.284	10.864	11.481	1.235
100.00%	66.543	68.272	66.914	10.864	21.358	1.235

**Note:** Resource Quality (RQ); Operational Quality (OQ); Personal Contact Quality (PQ); Information Quality (IQ); Customer Satisfaction (CS); Customization Quality (CQ); Re-use Intention (RI)

This study explored how OQ, RQ, IQ, OQ, and CQ, constituting LSQ components, influence CS, how CS influences logistics service RI, and whether the CS of LSQ mediates the connection between the elements of LSQ and logistics service RI. In this study, five components of the LSQ—OQ, RQ, IQ, PQ, and CQ—were used to determine the influence of the LSQ on customer satisfaction. Overall, 11 hypotheses were proposed and all relationships were found to be significant in the empirical investigation. The explanatory power of the exogenous constructs on the endogenous construct was also substantial, indicating that the model fit the investigation well. The following is a specific discussion of these relationships.

Hypothesis 1 posited that the operational quality of LSQ positively influences satisfaction. The results of this study support this hypothesis. This outcome is supported by the previous research conducted by Sorkun et al. [9]. They studied LSQ on omnichannel distribution for logistics services and identified that OQ influences the overall experience of the customer, from the order placement process to the delivery of goods. The faster and more accurately the logistics service provider can deliver goods, the greater the possibility of gaining CS via the services. Hence, LSP must emphasize OQ to enhance CS and achieve business success. Similar findings were revealed for Hypothesis 2, for which the results of this study claimed that customers are likely to be more satisfied with LSPs that have modern, well-maintained equipment. This includes everything from trucks and ships to warehouses and equipment handling. LSPs must have well-trained, experienced, and customer-focused staff that can promptly respond to and effectively address customers’ concerns and needs. This includes everyone from sales representatives and customer service personnel to drivers and warehouse workers. This result is consistent with early studies by Yeo et al. [52] and Gupta et al. [11]. This means that when logistics service providers invest in the latest technology, equipment, and employees, they can help ensure that goods are handled efficiently and effectively, thus reducing the likelihood of delays and damage during transportation. This, in turn, helps enhance CS by providing higher-quality services.

This study revealed that IQ offers a significant connection with CS for logistics services (H3). The scholars [10] supported this finding. However, the effect size of IQ on CS was relatively small compared to the other stimulus (LSQ) factors. This suggests that while IQ is a factor that contributes to CS, it falls short of being a necessary condition for the emergence of CS. IQ refers to the timeliness, accuracy, completeness, relevance, and clarity of information provided to customers. Logistics services rely heavily on the information exchange and communication between service providers and customers, making information quality a crucial factor in CS.

The outcome of this study suggests that PQ has a significant connection with CS in logistics services, supporting the hypothesis that PCQ directly affects CS (H4). As PQ increased, customers were more likely to be satisfied with their received logistics services, which is in accordance with previous studies [9, 10] that highlighted the importance of human factors in service delivery. Uvet [9] studied logistics service quality in the USA based on student views and found that PQ is a significant predictor of CS. These findings indicate that in addition to LSQ, the quality of personal interactions between customers and LSPs is a necessary factor in determining CS.

This study also found that CQ has a significant positive effect on CS for logistics services (H5). This outcome is in line with earlier research [10, 55] that showed that customization is a crucial factor in satisfying customer needs and preferences. Customization quality involves meeting customers’ specific requirements and tailoring services to their unique needs. When logistics service providers provide customized services to customers, they can increase the likelihood of CS, leading to loyalty and repeat business. The positive influence of CQ on CS suggests that LSPs should focus on offering customized solutions to meet customer needs. In doing so, they can differentiate themselves from their competitors and increase their value propositions to customers.

In addition, the findings for hypothesis (H6) illustrated that CS with logistics services is significantly linked with logistics service RI. This finding implies that if customers are satisfied with the logistics services they receive, they are more likely to reuse them in the future. This corroborates previous research [45] showing that CS is a significant predictor of customer RI. The findings suggest that satisfied customers are more likely to reuse logistics services, which can lead to increased customer loyalty and profitability for logistics service providers. One of the key takeaways from the study is the distinction between loyalty and reuse intention. While the two concepts are related, they are not interchangeable. Loyalty refers to the long-term commitment of a customer to a brand or company, which is often influenced by factors such as trust, emotional attachment, and perceived value. Conversely, reuse intention refers to a customer’s intention to use a specific service again in the future, which may or may not be influenced by loyalty.

By mediating analysis, the research hypotheses (H_M1_, H_M2_, H_M3_, H_M4_, and H_M5_) for testing the customer satisfaction of logistics services mediate the impact between the five components of LSQ and the re-use intention of logistics services. This study indicates that CS plays a significant role in mediating the link between operational quality, responsiveness, information quality, customization quality, personal contact quality, and logistics service RI. However, all the mediating relationships are partially mediated as all the relationships got significant. The positive and significant indirect effects of each of these quality dimensions and RI through CS suggest that enhancing customer satisfaction can lead to increased repeat business.

## Conclusion

In conclusion, this study provides valuable insights into the factors linking CS and RI in the logistics service sector. These findings suggest that the PQ, CQ, OQ, RQ, and IQ are significant predictors of CS. Furthermore, CS was found to mediate the connection between these quality dimensions and RI. The results indicate that LSP needs to emphasize improving its service quality, particularly in terms of personal contact and customization, to enhance customer satisfaction and encourage repeat business. The large sample size and rigorous analysis using PLS-SEM, NCA, and bottleneck analysis further enhanced the credibility of the findings.

This study makes the following theoretical contributions. *First*, it provides a comprehensive model that adds five dimensions of LSQ—operational quality, responsiveness quality, information quality, customization quality, and personal contact quality—to explain their impact on CS and RI in the logistics industry. *Second*, this study identified CS as a significant mediator in the connection between the LSQ dimensions and RI. Although several other studies have conducted mediation experiments with general SQ dimensions (such as tangibility, responsiveness, empathy, assurance, and reliability), this study established the mediation effect of satisfaction within the LSQ dimensions and RI, providing significant insights into the logistics literature. This finding substantiates the literature by providing a thorough understanding of how the LSQ dimensions impact customer re-use intentions through the mediating effects of CS.

*Third*, this study provides empirical evidence of the effect of LSQ dimensions on RI in the logistics service arena from the perspective of a developing country (China), which expands the existing literature on SQ in the logistics industry. In addition, by integrating the reuse intentions of logistics services, this study acknowledges the limitations of Sorkun et al. [9]. *Finally*, the study also used a large sample size and a structural equation modeling approach, which further enhanced the credibility of the findings and helped in a more comprehensive understanding of the relationships. The NCA provided a clear picture of how various factors affect the outcome, and bottleneck analysis helped determine the predictors that have the greatest impact on the outcome and are critical to improvement.

The outcomes of this research offer practical implications for logistics service providers seeking to improve customer satisfaction and increase service re-use intentions. *First*, they must prioritize improving their OQ and IQ, which have a direct positive influence on CS. Specifically, they should pay attention to aspects such as timely and accurate delivery, effective and efficient problem-solving, clear and comprehensive information, and courteous and friendly communication. Moreover, LSP can use the outcomes of this study to finalize the specific dimensions of LSQ that have the greatest effect on CS and prioritize their efforts accordingly. Particularly, LSPs should invest in improving the quality of information they provide to customers to increase their satisfaction, trust, and loyalty. This may involve the development of user-friendly platforms and interfaces, providing up-to-date and accurate information, and improving communication channels. By doing so, logistics service providers can gain competitive competence in the market and increase their customer base.

The results of this study show that PQ is an important predictor of CS in logistics services. To enhance PQ, logistics service providers can focus on several key areas, such as implementing customer relationship management systems, which can help staff better manage customer interactions and provide personalized services. Customer-facing staff can be trained on effective communication, and interpersonal skills can help them better understand customer needs and preferences and provide more personalized services. In addition, providing incentives and recognition to staff for providing excellent customer service can motivate them to deliver higher-quality service. Based on this analysis, enhancing customization quality can improve customer satisfaction with logistics services. To enhance customization quality, logistics companies can focus on providing personalized services and tailoring their offerings to meet individual customer needs. Companies can offer flexible and customizable delivery options, such as same-day or time-specific deliveries, to meet customer expectations and improve their overall experiences. Moreover, logistics service providers should hire skilled employees and provide them with adequate training and resources to support personal interactions.

*Second*, this study highlights the importance of customer satisfaction as a significant predictor of service re-use intentions. Thus, LSPs must ascertain that their customers are satisfied with the services delivered and that they meet their needs and expectations. This can be achieved through continuous monitoring and improvement of service quality as well as regular communication with customers to understand their preferences and concerns. By improving CS, LSPs can improve their reputation, attract new customers, and increase their revenue. *Finally*, the study reveals that CS mediates the relationships between operational quality, information quality, responsiveness, assurance, empathy, and service reuse intentions. This implies that logistics service providers should concentrate on improving CS by enhancing these service quality dimensions, as this will result in higher RI. Thus, they can establish a loyal customer base and gain competitive competence in the market.

## Limitations and future research directions

*First*, this study, being solely quantitative, may lack the comprehensive understanding that can be gained through a qualitative research design. For example, results obtained in a highly controlled environment may not necessarily translate to a real-life setting. Thus, mixed methods may be suggested for future studies. *Second*, in this study, a sample of customers was drawn from a single developing economy, which may not be generalizable to all economies. To expand the scope of this research, future studies could explore other contexts, particularly in developed countries that have more established experience with logistics service delivery.

*Third*, this study was restricted to customers who had shopped earlier at a specific retailer, which may have resulted in a biased sample. Even though a mean CS score of 5.26 out of 7 indicates a limited amount of bias, there is still a possibility of some bias in the results. One solution to avoid this limitation may be to seek responses based on the latest shopping experiences. Nevertheless, this may lead to a tradeoff between bias and reliability. Advanced research is recommended to develop approaches that can overcome this bias-reliability trade-off. *Fourth*, CS represents only a single performance variable that affects service quality and was used in the current study. Although it considers repurchase intention, to enhance future research, additional performance constructs, such as word-of-mouth and loyalty, should be taken into account to diversify their impacts. *Fifth*, the model mentioned in this study did not consider moderating variables such as demographic factors including age, sex, occupation, and monthly income. These factors may affect logistics service customers’ decision-making. Future research may consider including moderating factor variables to investigate how demographic characteristics, such as sex, age, and so on, influence perceptions of customer satisfaction or logistics service re-use intention.

## Supporting information

S1 File Survey questionnaire. (DOCX)

S2 File Dataset. (CSV)

S1 Checklist (DOCX)

S1 Data (CSV)
