# Supplementary material for: Examining the effect of logistics service quality on customer satisfaction and re-use intention
Source: PLoS One. 2023 May 31;18(5):e0286382. doi: 10.1371/journal.pone.0286382 (PMC10231832; doi:10.1371/journal.pone.0286382)
Supplement: S1 File — (DOCX) [file pone.0286382.s001.docx]

***Supporting Material S1.*** Survey Questionnaire

| Code | Questions |
| --- | --- |
| *Operational Quality (OQ)* | |
| OQ1 | Delivers the right product to the right place. |
| OQ2 | Having sufficient temporary or permanent storage capacity. |
| OQ3 | Delivers the promised services without failure. |
| OQ4 | Delivers the goods without loss or damage. |
| OQ5 | Handles trade documents effectively. |
| *Resource Quality (RQ)* | |
| RQ1 | Availability of physical facilities. |
| RQ2 | Availability of an adequate workforce. |
| RQ3 | Geographical reach and network coverage. |
| RQ4 | The fleet and physical facilities are modern and always function properly. |
| RQ5 | Adequately maintained and updated information technology. |
| *Information Quality (IQ)* | |
| IQ1 | Information that communicated by the LSP is complete. |
| IQ2 | Information that communicated by the LSP is timely. |
| IQ3 | Information that communicated by the LSP is accurate. |
| IQ4 | Information that communicated by the LSP is adequate. |
| IQ5 | Information that communicated by the LSP is credible. |
| *Personal Contact Quality (PQ)* | |
| PQ1 | Contact employees to make an effort to understand my situation. |
| PQ2 | Contact employees can resolve product/service problems. |
| PQ3 | The service knowledge/experience of contact employees is sufficient/adequate. |
| PQ4 | Shows a positive attitude while dealing with my situation. |
| PQ5 | Maintains courteous behaviour while dealing. |
| *Customization Quality (CQ)* | |
| CQ1 | The logistics service provider offers services for customers’ requests. |
| CQ2 | The logistics service provider offers me different options for logistics services. |
| CQ3 | The logistics service provider offers me services that satisfy my specific needs. |
| CQ4 | The logistics service provider offers me services that I couldn’t find in other logistics providers. |
| CQ5 | If I changed between companies, I wouldn’t obtain services as customized as I have now. |
| *Customer Satisfaction (CS)* | |
| CS1 | I am delighted with the performance of my logistics service provider. |
| CS2 | The services offered by the logistics service provider meet my expectations. |
| CS3 | The services provided to you through the logistics service provider are good. |
| CS4 | Overall, I am satisfied with my logistics service provider. |
| CS5 | My feelings towards the logistics service provider are very positive. |
| CS6 | I feel good about choosing this logistics service provider for the offering I am looking for. |
| *Re-use Intention (RI)* | |
| RI1 | Willing to use a logistics service provider in the future. |
| RI2 | Use logistics service providers when relevant information is needed. |
| RI3 | Willing to recommend the logistics service provider to others. |
| RI4 | Explain the positive aspects of a logistics service provider to others. |
| RI5 | I will recommend the logistics service provider to the people around me. |

**Note:** Resource Quality (RQ); Operational Quality (OQ); Personal Contact Quality (PQ); Information Quality (IQ); Customer Satisfaction (CS); Customization Quality (CQ); Re-use Intention (RI)

***Supporting Material 2.*** Loading, Cross-Loading and Fornell-Larcker criterion

|  | OQ | RQ | IQ | PQ | CQ | CS | RI |  |
| --- | --- | --- | --- | --- | --- | --- | --- | --- |
| OQ1 | *0.921* | 0.310 | 0.294 | 0.189 | 0.084 | 0.310 | 0.042 |  |
| OQ2 | *0.936* | 0.302 | 0.315 | 0.227 | 0.095 | 0.318 | 0.047 |  |
| OQ3 | *0.933* | 0.319 | 0.350 | 0.250 | 0.155 | 0.344 | 0.058 |  |
| OQ4 | *0.944* | 0.314 | 0.322 | 0.236 | 0.153 | 0.336 | 0.070 |  |
| OQ5 | *0.929* | 0.289 | 0.334 | 0.225 | 0.129 | 0.333 | 0.053 |  |
| RQ1 | 0.353 | *0.881* | 0.436 | 0.418 | 0.407 | 0.466 | 0.483 |  |
| RQ2 | 0.202 | *0.873* | 0.398 | 0.416 | 0.416 | 0.450 | 0.481 |  |
| RQ3 | 0.298 | *0.875* | 0.442 | 0.408 | 0.407 | 0.477 | 0.443 |  |
| RQ4 | 0.272 | *0.854* | 0.415 | 0.377 | 0.388 | 0.467 | 0.441 |  |
| RQ5 | 0.308 | *0.883* | 0.438 | 0.386 | 0.419 | 0.485 | 0.480 |  |
| IQ1 | 0.357 | 0.443 | *0.881* | 0.435 | 0.400 | 0.452 | 0.453 |  |
| IQ2 | 0.261 | 0.433 | *0.893* | 0.439 | 0.423 | 0.471 | 0.525 |  |
| IQ3 | 0.282 | 0.436 | *0.868* | 0.420 | 0.407 | 0.508 | 0.473 |  |
| IQ4 | 0.297 | 0.425 | *0.874* | 0.446 | 0.419 | 0.517 | 0.503 |  |
| IQ5 | 0.327 | 0.403 | *0.867* | 0.399 | 0.403 | 0.486 | 0.458 |  |
| PQ1 | 0.160 | 0.374 | 0.413 | *0.844* | 0.481 | 0.464 | 0.491 |  |
| PQ2 | 0.153 | 0.388 | 0.419 | *0.852* | 0.497 | 0.419 | 0.531 |  |
| PQ3 | 0.210 | 0.363 | 0.405 | *0.851* | 0.440 | 0.461 | 0.489 |  |
| PQ4 | 0.223 | 0.401 | 0.405 | *0.856* | 0.437 | 0.478 | 0.531 |  |
| PQ5 | 0.282 | 0.424 | 0.433 | *0.847* | 0.500 | 0.458 | 0.475 |  |
| CQ1 | 0.236 | 0.440 | 0.444 | 0.485 | *0.823* | 0.439 | 0.542 |  |
| CQ2 | 0.087 | 0.362 | 0.385 | 0.428 | *0.833* | 0.417 | 0.499 |  |
| CQ3 | 0.113 | 0.388 | 0.411 | 0.446 | *0.814* | 0.350 | 0.530 |  |
| CQ4 | 0.022 | 0.370 | 0.360 | 0.439 | *0.845* | 0.381 | 0.538 |  |
| CQ5 | 0.076 | 0.364 | 0.328 | 0.490 | *0.818* | 0.361 | 0.531 |  |
| CS1 | 0.321 | 0.475 | 0.476 | 0.491 | 0.387 | *0.801* | 0.470 |  |
| CS2 | 0.278 | 0.499 | 0.454 | 0.391 | 0.379 | *0.821* | 0.496 |  |
| CS3 | 0.282 | 0.428 | 0.475 | 0.468 | 0.400 | *0.849* | 0.540 |  |
| CS4 | 0.286 | 0.452 | 0.428 | 0.447 | 0.366 | *0.833* | 0.504 |  |
| CS5 | 0.243 | 0.414 | 0.463 | 0.427 | 0.444 | *0.824* | 0.511 |  |
| CS6 | 0.334 | 0.381 | 0.452 | 0.426 | 0.367 | *0.808* | 0.441 |  |
| RI1 | 0.127 | 0.470 | 0.497 | 0.503 | 0.558 | 0.526 | *0.845* |  |
| RI2 | 0.030 | 0.460 | 0.480 | 0.493 | 0.558 | 0.524 | *0.836* |  |
| RI3 | 0.009 | 0.443 | 0.453 | 0.504 | 0.507 | 0.517 | *0.847* |  |
| RI4 | -0.005 | 0.424 | 0.442 | 0.490 | 0.541 | 0.453 | *0.838* |  |
| RI5 | 0.079 | 0.460 | 0.461 | 0.519 | 0.544 | 0.522 | *0.874* |  |
| *Fornell-Larcker criterion* | | | | | | | | |
| OQ | 0.933 |  |  |  |  |  |  |  |
| RQ | 0.329 | 0.873 |  |  |  |  |  |  |
| IQ | 0.347 | 0.488 | 0.877 |  |  |  |  |  |
| PQ | 0.243 | 0.459 | 0.488 | 0.850 |  |  |  |  |
| CQ | 0.133 | 0.467 | 0.468 | 0.553 | 0.827 |  |  |  |
| CS | 0.352 | 0.537 | 0.557 | 0.537 | 0.475 | 0.823 |  |  |
| RI | 0.058 | 0.533 | 0.551 | 0.592 | 0.639 | 0.601 | 0.848 |  |

**Note:** Resource Quality (RQ); Operational Quality (OQ); Personal Contact Quality (PQ); Information Quality (IQ); Customer Satisfaction (CS); Customization Quality (CQ); Re-use Intention (RI)
