# Supplementary material for: Examining the effect of logistics service quality on customer satisfaction and re-use intention
Source: PLoS One. 2023 May 31;18(5):e0286382. doi: 10.1371/journal.pone.0286382 (PMC10231832; doi:10.1371/journal.pone.0286382)
Supplement: S1 Checklist — (DOCX) [file pone.0286382.s003.docx]

***PLOS ONE* Clinical Studies Checklist**

***PLOS ONE* manuscript number: _______________________**

| **Complete the following if your study involved human participants or human subjects’ data. These questions should be addressed for prospective and retrospective studies.** | | |
| --- | --- | --- |
| 1. | Did you obtain ethics approval for this study?   - If yes, please upload (file type “Other”) the original approval document you received from your ethics committee. If the original document is in another language, please also provide an English translation.   ___ Uploaded X N/A   - If you did not obtain ethical approval, please explain why this was not required.  \| According to the "Ethical Review of Biomedical Research Involving Human Beings," a public source from China's National Health and Wellness Commission, all life science and medical research activities involving human beings should be subject to ethical review (<http://www.gd.gov.cn/zwgk/wjk/zcfgk/content/post_2530813.html>). Business research and management are not part of life science and medical research activities involving human beings and only require clear and explicit informed consent prior to data collection. No formal ethics approval was therefore required in this particular case because (a) the data is completely anonymous with no personal information being collected; (b) the data is not considered to be sensitive or confidential in nature; (c) the issues being researched are not likely to upset or disturb participants; (d) vulnerable or dependent groups are not included; and (e) there is no risk of possible disclosures or reporting obligations. This study has been performed in accordance with the Declaration of Helsinki. Written informed consent for participation was obtained from respondents who participated in the survey. For the respondents who participated in the survey were asked to read the ethical statement posted at the top of the form (*There is no compensation for responding, nor is there any known risk. In order to ensure that all information will remain confidential, please do not include your name. Participation is strictly voluntary and you may refuse to participate at any time*) and proceed only if they agree. No data was collected from anyone under 18 years old. \| \| --- \| |  |
| 2. | If your study involved human participants, please report in the Methods section when participants were recruited to the study.  _X_ Completed _ N/A |  |
| 3. | If you are reporting a study of medical records or archived samples, please report in the Methods section the date range in which human subjects’ data/samples were collected and the date(s) when you conducted this study.  X_ Completed _ N/A |  |
| 4. | Please specify in the Methods section whether authors had access to information that could identify individual participants during or after data collection.  X_ Completed N/A |  |
| 5. | If you are reporting an observational study – i.e. cohort, case-control, and cross-sectional studies – we recommend that the work is reported as per the requirements of the STROBE guidelines, and that you provide a completed STROBE checklist as a Supporting Information file with your submission.  The STROBE checklist was developed to improve the reporting of observational human subjects research, and is available here: <http://strobe-statement.org/fileadmin/Strobe/uploads/checklists/STROBE_checklist_v4_combined_PlosMedicine.docx>.  ___ Completed x N/A |  |
| 6. | Please ensure that the author list and Corresponding Author entered in Editorial Manager match the author list and Corresponding Author in your manuscript file.  x_ Completed |  |
